# Introducing the kollaR package: A user-friendly open-access solution for eye-tracking analysis and visualization

**DOI:** 10.3758/s13428-025-02903-z

**Published:** 2025-12-08

**Authors:** Johan Lundin Kleberg, Astrid E. Z. Hallman, Rebecka Astenvald, Ann Nordgren, Terje Falck-Ytter, Ronald van den Berg

**Affiliations:** 1 Department of Psychology, Stockholm University, Frescativägen 8, 106 91 Stockholm, Sweden; 2 Department of Clinical Neuroscience, Karolinska Institutet, Stockholm, Sweden; 3 Department of Medical Sciences, Uppsala University, Uppsala, Sweden; 4 Department of Molecular Medicine and Surgery, Karolinska Institutet, Stockholm, Sweden; 5 Department of Clinical Genetics and Genomics, Karolinska University Hospital, Stockholm, Sweden; 6 Department of Clinical Genetics and Genomics, Sahlgrenska University Hospital, Gothenburg, Sweden; 7 Region Västra Götaland, Gothenburg, Sweden; 8 Institute of Biomedicine, Department of Laboratory Medicine, University of Gothenburg, Gothenburg, Sweden; 9 Development and Neurodiversity Lab, Department of Psychology, Uppsala University, Uppsala, Sweden; 10 Center of Neurodevelopmental Disorders (KIND), Centre for Psychiatry Research, Department of Women’s and Children’s Health, Karolinska Institutet & Stockholm Health Care Services, Stockholm, Sweden

**Keywords:** Eye tracking, R Package, Fixations, Saccades, Software, Area of Interest

## Abstract

Eye tracking has become an increasingly important tool in cognitive and developmental research, providing insights into processes that are difficult to measure otherwise. The majority of eye-tracking studies rely on accurate identification of fixations and saccades in raw data using event classification algorithms (sometimes called fixation filters). Subsequently, it is common to analyze whether fixations or saccades fall into specific areas of interest (AOI). The choice of algorithms can significantly influence study outcomes, especially in special populations such as young children or individuals with neurodevelopmental conditions, where data quality is often compromised by factors such as signal loss, poor calibration, or movement artifacts. It is therefore crucial to examine how available fixation classification algorithms affect the data set at hand as part of the eye-tracking analysis. Here, we introduce the kollaR package, an open-source R library for performing the main steps of an eye-tracking analysis from event classification to AOI-based analyses and visualizations of individual or group-level data for publications. The kollaR package was specifically designed to facilitate the selection and comparison of different event classification algorithms through visualizations. In a validation analysis, we show that results from fixation classification in kollaR are consistent with those from other software implementations of the same algorithms. We demonstrate the use of kollaR with real data from typically developing individuals and individuals with neurodevelopmental conditions, and illustrate how potential threats to validity can be identified in both high- and low-quality data.

## Introduction

The use of eye tracking in research is rapidly expanding. Thanks to its high temporal and spatial resolution and relative ease of administration, eye tracking can provide insights into cognitive processes that are otherwise unavailable to researchers. This may be especially valuable in studies including young children or participants with limited verbal ability. Consequently, it has become a popular tool in research which examines topics such as the earliest signs of autism (Del Bianco et al., 2020; Elsabbagh & Johnson, 2016; Falck-Ytter et al., 2023), social perception in rare genetic conditions (Kleberg et al., 2022; Riby & Hancock, 2008; Vivanti et al., 2016), attention in children with autism and ADHD (Del Bianco et al., 2020; Keehn et al., 2023; Kleberg et al., 2023; Lockwood Estrin et al., 2024) and various aspects of infant development (e.g., Falck-Ytter et al., 2023; Viktorsson et al., 2023). Recent studies indicated a possible clinical application for early detection of autism (Jones et al., 2023).

To make sense of eye-tracking data, it is crucial to distinguish valid gaze location measurements from invalid ones caused by movement artifacts, blinks, and equipment imprecision. The majority of eye-tracking analyses also depend on the identification of *fixations* and often also *saccades* (Duchowski, 2017). As an operational oculomotor definition, fixations are periods of relatively stable gaze, typically ranging from 150 to 300 ms. In contrast, saccades are rapid ballistic eye movements occurring between fixations (for a discussion of definitions of fixations and saccades, see Hessels et al., 2018). Fixated areas of the visual field are highly prioritized in information processing, whereas little cognitive processing of visual information takes place during saccades. Data from infants, young children with autism, and other neurodevelopmental conditions are typically noisy and require special consideration of the analytic pipeline to avoid invalid conclusions (Kylliäinen et al., 2014).

Fixations and saccades are typically identified using event classification algorithms (Salvucci & Goldberg, 2000), sometimes also referred to as fixation filters (e.g., Olsen, 2012). Although it has long been known that the choice of event classification algorithms and their parameters can have large effects on the results of eye-tracking studies (Andersson et al., 2017; Birawo & Kasprowski, 2022; Hooge et al., 2022; Startsev & Zemblys, 2023), both are often insufficiently described in published research, even in highly influential publications (for a detailed recommendation for reporting event classification, see Dunn et al., 2023). Data with high levels of noise, which is common in clinical populations, is particularly sensitive to algorithm choices (Hessels et al., 2017; Shic et al., 2008; Wass et al., 2014). In an extreme example, Shic and colleagues described how the choice of a suboptimal method could lead to a reversal of an expected effect in a study comparing children with autism to neurotypical controls (Shic et al., 2008). To reduce the risk of incorrect results, researchers are recommended to carefully visualize and evaluate how potential event classification algorithms affect their results.

Most eye-tracker manufacturers sell software together with their hardware, which can be used for event classification. While this can sometimes simplify analyses, it can also make it difficult for researchers to evaluate the effects of specific algorithms and their parameters. In some cases, manufacturers do not report the exact calculations underlying fixation and saccade classification in their software (Andersson et al., 2017; Niehorster et al., 2025), making important steps of the research process difficult to evaluate.

Research questions, variables of interest, and study design will partly determine the type of threats to validity that may have to be addressed. For example, superior visual search by children with autism has been associated with either shorter average fixations (Joseph et al., 2009, but see Keehn et al., 2023) or a reduced number of fixations overall (Keehn et al., 2023). Studies making such claims are only valid if the chosen event classification procedure can accurately detect fixations and their on- and offsets. In many studies, the main variable of interest is the latency to initiate or complete a gaze shift. For example, several studies reported delayed orienting of gaze in neurodevelopmental conditions, including autism, ADHD, and rare genetic conditions (Elsabbagh et al., 2013; Kleberg et al., 2023; Sabatos-Devito et al., 2016). For these claims to be valid, it is crucial that the analysis can correctly differentiate between gaze shifts, noise, and other events such as fixations. In any study reporting group comparisons, a crucial assumption is that the performance of the analysis methods does not vary with group membership, for example, that a higher number of fixations is not detected in autistic than non-autistic children simply because the former group has more noisy data.

Notably, the research question, study design, and participant population will also affect the desired properties of event classification algorithms and the analytic pipeline in general. For example, researchers may be interested in eye movement metrics such as saccade onset or peak velocity in their own right. In this case, it is important to evaluate whether the results of event detection algorithms match known physiological properties of these events. In other studies, fixations serve as an indirect proxy for visual attention. Here, it is essential to differentiate between fixations and noise or lost data, but it may be less important to determine the exact number of fixations (Hessels et al., 2024; Hooge et al., 2025). There are also special situations where researchers may choose not to classify fixations at all, but instead analyze the aggregated number of samples that fall into specific areas of interest (AOIs). These include infant studies that examine preferential looking at one or more large AOIs over time periods of several seconds. Given the challenges with fixation classification in infant data (Wass et al., 2013), researchers conducting this type of study may prefer to analyze all recorded gaze samples, regardless of whether they fall into fixations or not (Rudling et al., 2024; Viktorsson et al., 2023). For discussions of fixation classification in infant data, see Wass et al., 2013; Hessels et al., 2017. To sum up, no procedure for classifying fixations and saccades will be optimal in every research setting, and possible analytic choices must be evaluated in light of the data and the research question at hand.

## The kollaR package

Here, we introduce the kollaR package,^1^ an R package for analyzing eye tracking data, comparing event classification algorithms, and visualizing the results for quality control, presentations or publications. These functions are meant to provide researchers with a freely available and simple tool for applying and evaluating event classification algorithms on their own eye tracking data. kollaR is meant to facilitate analyses and provide a set of tools for evaluating the effects of methodological choices, but will not replace researcher judgment in designing an analysis pipeline. We refer to recent tutorials and guidelines for in-depth discussions about eye-tracking study design and analyses (Duchowski, 2017; Dunn et al., 2023; Hessels & Hooge, 2019; Hessels et al., 2024; Hooge et al., 2022, 2025). In the following sections, we introduce key concepts of eye-tracking analyses, describe kollaR, and compare its fixation classification with that of other software. After this, we demonstrate how visualization and comparison of event classification algorithms in real data from individuals with neurodevelopmental conditions can identify potential threats to data validity and help researchers to address them.

## Error and noise in eye-tracking data

Movement artefacts, blinks, technical problems with equipment and setup, and, in some cases, variation in eye anatomy affect the quality of eye-tracking data. Problems with data quality can be of several forms. Eye-tracking data will typically contain periods in which gaze coordinates are missing altogether (data loss), operationalized as the proportion of missing gaze coordinates during a recording or a part of the data (Hessels & Hooge, 2019). A second source of error is *low spatial accuracy*, or systematic differences between the true and recorded gaze position. Unlike data loss, which can be assessed directly in the data, spatial accuracy requires a known true gaze position. *Imprecision* can be defined as variability in consecutive gaze position measurements in the absence of actual eye movements. It is typically measured as the sample-to-sample root-mean-square distance (RMSD). Lower RMSD values reflect lower variability and thus higher precision (Dunn et al., 2023; Hessels & Hooge, 2019; Holmqvist et al., 2012).

Excessive sample-to-sample variability is common in eye-tracking data from young children and individuals with neurodevelopmental conditions (Hessels & Hooge, 2019; Shic et al., 2008; Wass et al., 2014). This can stem from several sources, including eye tracker imprecision, poor calibration, and movement artefacts. Excessive variability can, in turn, affect event detection. For example, Wass et al. (2013) found that higher sample-to-sample variability in infant data could lead to apparently shorter duration of classified fixations. The following sections will mainly address questions of data validity related to data loss and variability in gaze position estimates.

## Event classification algorithms

A number of event classification algorithms have been proposed. The identification by dispersion threshold (I-DT) algorithm (Salvucci & Goldberg, 2000) classifies time segments as fixations when a sufficient number of subsequent samples are within a spatially restricted area defined by a threshold parameter, typically the radius of a circle. Subsequent samples are included in a candidate fixation as long as their Euclidean distance from the centroid of the included samples does not exceed the threshold. A threshold of 1° of the visual field has been recommended (Blignaut, 2009), which aligns with the fact that the fovea covers approximately 2° of the visual field (Duchowski, 2017). To avoid physiologically unlikely short periods in the data being classified as fixations, a temporal threshold is also defined. Saccades can then be defined as periods occurring between fixations.

**Fig. 1:**
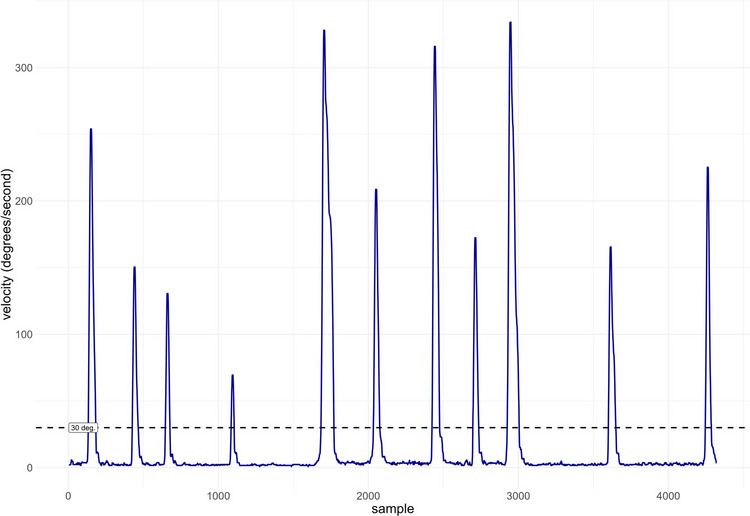
Sample-to-sample velocity in eye-tracking data recorded at 1200 Hz during approximately 2 s. The *dashed horizontal line* shows a commonly used threshold for saccade identification of 30 degrees per second. The participant is scanning a complex image. The figure was generated with the kollaR function plot_sample_velocity

While the I-DT algorithm classifies events in the data by identifying fixations, the identification-by-velocity-threshold (I-VT) algorithm (Salvucci & Goldberg, 2000) instead starts by classifying data periods as saccades based on sample-to-sample velocity. Velocity can be computed as the Euclidean distance between two samples divided by the between-samples time interval. Figure 1 illustrates the sample-to-sample velocity during a 2-s interval of data recorded at 1200 Hz. As can be seen, periods of relatively stable gaze position (i.e., low velocity) are interrupted by rapid increases in velocity. The I-VT algorithm classifies periods of velocity exceeding a specified threshold as belonging to a saccade and periods between them as fixations. Thresholds ranging from 20 to 40 degrees per second have been suggested (Olsen & Matos, 2012; Salvucci & Goldberg, 2000). It should be noted that operationalization of fixations as periods in between saccades is a simplification which may be problematic, for example, when moving objects are tracked across the visual field (e.g., smooth pursuit, Duchowski, 2017).

The data shown in Fig. 1 contain a number of saccade candidates that would all be detected using the I-VT algorithm with a threshold of 30 degrees per second. The choice of threshold can be guided by theoretical considerations or visual inspection of the data, but it is inherently somewhat subjective. Moreover, a single fixed threshold is unlikely to generalize across datasets, as the optimal value can depend on factors such as noise levels and calibration quality. Alternative algorithms therefore determine threshold values based on properties of the data, particularly the level of noise (Engbert & Kliegl, 2003; Nyström & Holmqvist, 2010). This flexible approach allows separate thresholds for specific participants and even trials. The Nyström and Holmqvist (2010) *adaptive threshold algorithm* starts by identifying peak velocities in the data. A peak is identified when gaze velocity exceeds a threshold defined as $\overline{\text{v }+\uplambda {\mathrm{s}}_{\mathrm{v}}}$, where $\overline{\mathrm{v} }$ is the average velocity, s_v_ is the standard deviation of velocity measurements, and λ a parameter that controls how conservative the algorithm is (typically set to 6). A peak is identified when gaze velocity exceeds a peak velocity threshold defined as mean sample-to-sample velocity + Λ times its standard deviation, where Λ is a parameter. The peak velocity threshold is then iteratively updated based on information about the sample-to-sample changes in velocity of all samples under this threshold. Thus, the threshold value is adapted to the overall noise in the data.

Once a threshold for peak saccadic velocities has been determined, the algorithm searches backwards from each peak in the velocity data to determine the onset of each saccade, and forwards to determine its offset. On- and offset are determined by velocity thresholds that are set based on weighting the peak velocity threshold and the level of noise in the data. The exact calculations of the adaptive velocity threshold algorithm are described in (Nyström & Holmqvist, 2010) and discussed elsewhere (Andersson et al., 2017; Hooge et al., 2022; Niehorster et al., 2015, 2025).

Several studies have evaluated and compared versions of velocity- and dispersion-based algorithms and compared their performance at different threshold values (Blignaut, 2009; Olsen & Matos, 2012; Salvucci & Goldberg, 2000). Versions of and combinations of the two algorithm families are reviewed by Andersson et al. (2017) and Hein and Zangemeister (2017).

Hessels and colleagues proposed the Identification by Two Means Clustering (I2MC) algorithm to deal with data with high levels of noise and data loss. This algorithm iteratively selects data within a sliding time window (with a default length of 200 ms). Within this time window, gaze samples are grouped into two separate clusters based on their distance from the cluster centroid using k-means clustering. According to the logic of the I2MC algorithm, time windows, which include a transition from one fixation to another, should be characterized by two clearly distinguishable data clusters and a transition between them. The on- and offsets of the time window are shifted in small steps, so that each sample is included in the k-means clustering period multiple times. The procedure is then repeated in down-sampled data. Following this, fixations are defined as periods between reliably identified transitions (for a full description, see Hessels et al., 2017). The I2MC algorithm was found to be more robust to noise and missing data than less complex algorithms such as I-VT and I-DT (Hessels et al., 2017; Hooge et al., 2022). For a full description of the algorithm, see Hessels et al., (2017).

## Setting a threshold value for fixations and saccades

It is advisable to complement the event classification algorithm with rules for discarding unlikely short fixations and saccades. For example, many event classification algorithms merge fixations that are close in space (e.g., within 0.7 degrees and time (e.g., 50 ms). It is important to report procedures for handling short fixations and saccades, as they can sometimes have a higher impact on the results than the choice of an algorithm per se. For example, Hooge et al. (2022) compared a range of fixation classification algorithms and found similar performance in high-quality data as long as the rules for discarding fixations and saccades were the same (saccade amplitudes < 1 degree and fixation durations < 60 ms were recommended by Hooge et al., 2022).

Importantly, no single event classification will be optimal for all studies. Instead, it is crucial to evaluate for each individual data set how results are affected by different choices in the analysis pipeline (Duchowski, 2017; Hessels et al., 2017; Olsen & Matos, 2012). Factors affecting the performance of event classification algorithms include the sampling rate of the recorded data, the equipment, and the overall proportion of data loss resulting from movement artefacts and blinks. It is also important to consider the research question and variables of interest. For recent reviews and tutorials on these topics, see (Hessels et al., 2024; Hessels & Hooge, 2019; Hooge et al., 2025; Niehorster et al., 2025). In the next sections, we present an overview of the kollaR package before exemplifying how the included functions can be used to analyze real data from participants with intellectual disability.

## Overview of the kollaR package

The kollaR package includes functions for analyses of eye-tracking data from pre-processing to event classification and visualizations. An overview of the functions is presented in Table 1. A typical analysis will go from pre-processing to event classification, followed by area of interest (AOI)-based analyses. After this, researchers may want to create visualizations suitable for presentations and publication. However, the functions can be used in various orders and combined with other software, as long as the data follows a basic structure. kollaR works with any data set that includes *X* and *Y* gaze coordinates, timestamps for each coordinate, and which can be read into R as a data frame, for example,.csv or.xlsx files. The pre-processing, event-classification, and post-processing functions (see Table 1) require data at the single-sample level. The AOI-based analysis and fixation visualization functions work with single sample or fixation data in formats described in the documentation for each function. For example, it is possible to use the visualization functions to evaluate the performance of event classification algorithms implemented in manufacturer software such as Tobii Pro Lab or Tobii Studio (Tobii Inc., Danderyd, Sweden).

**Table 1:** Overview of functions in the kollaR package

Function	Purpose
Pre-processing before event classification	
preprocess_gaze	Pre-process data (smoothing and interpolation)
downsample_gaze	Down-sampling gaze data to a specified sampling rate
Event classification	
algorithm_adaptive	Adaptive velocity threshold algorithm (Nyström & Holmqvist, 2010)
algorithm_idt	Dispersion-based fixation classification algorithm
algorithm_ivt	Velocity-based fixation and saccade classification algorithm
algorithm_i2mc	Identification of fixations by a two-means clustering algorithm
Post-processing after event classification	
merge_adjacent_fixations	Merge fixations close in time and space (this is also an option in the event classification functions)
trim_fixations	Remove samples close to the onset and offset of a fixation, which may belong to a saccade
Visualization functions for comparison of event classification algorithms	
fixation_plot_2d	Plot individual samples overlaid on fixations from one or more algorithms in 2D space
fixation_plot_temporal	Plot the *X* or *Y* coordinates against time. Two time series can be plotted, for example, raw and pre-processed gaze position, or fixation coordinates and single samples
fixation_plot_ts	Plot the raw and fixation classified X or Y coordinates against time or sample numbers for multiple fixation classification algorithms
plot_sample_velocity	Plot the sample-to-sample velocity for a given time segment
plot_velocity_profiles	Plot the velocity profiles of saccades identified with the I-VT algorithm
plot_algorithm_results	Plot validity measures of fixations detected by one or more event classification algorithms
Visualization of gaze data after event classification	
static_plot	Create a static 2D plot of one or more participants’ fixations during a specified time segment. Fixations can be plotted on top of stimulus images
animated_fixation_plot	Create an animated.gif 2D plot of one or more participants’ fixations during a specified time segment. Fixations can be plotted on top of stimulus images
Area of interest (AOI) analysis	
draw_aois	Draw rectangular or elliptical AOIs on a stimulus image and save them for further analyses
aoi_test	Test whether fixations or saccades fall into one or several AOIs. The function returns total number of fixations, the accumulated fixation time, latency to first fixation inside and outside each AOI

Gaze coordinates in eye-tracking data may be expressed in degrees, screen proportion, or pixels, depending on the recording system. While degrees and milliseconds are useful for physiological interpretation or publication, other units can be more effective for validating algorithms or inspecting specific data segments. To accommodate these different analysis goals, kollaR supports flexible visualization across units.

### Installation of kollaR

kollaR is available in the CRAN repository. It can be installed on R by typing the command:

install.packages(“kollaR”)

### Differences between kollaR and existing packages

A number of other R packages for eye tracking analyses exist. The *Gazepath* package (van Renswoude et al., 2018) applies a data-driven velocity-based algorithm for fixation detection with a threshold that is automatically adapted to data quality. The *EyetrackingR* (Forbes et al., 2023) and *eyeTrackR* (Godwin & Muhl-Richardson, 2019) and *GazeR* (Geller et al., 2020) packages do not perform pre-processing and fixation classification but include sophisticated functions for sorting data and conducting analyses after these steps. The *emov* package (Schwab, 2016) implements the I-VT and I-DT algorithms but does not include functions for the visualization of their output for validation. For an overview of existing software packages in other programming languages, see (Niehorster et al., 2025).

## Comparing fixation classification in kollaR and other software: A validation analysis

To test the validity of fixation classification in kollaR, we compared the outputs of event classification functions to published software implementing the same algorithms. The following sections present descriptive statistics of the fixations classified by kollaR and existing software. To examine potential differences between algorithms in timing of fixations, we calculated the relative timing offset (RTO) and relative timing deviation (RTD) of fixation onsets and offsets between the fixations detected by kollaR and the comparison algorithm. For each fixation in kollaR, a target fixation in the comparison data was defined as the first fixation with an onset and offset within the target fixation interval, with a margin of 15 ms. Following Hooge et al. (2018), RTO of fixation onset (RTO_onset_) was defined as the average difference between the onsets, and RTD_onset_ as their standard deviation. Analogously, RTO_offset_ and RTD_offset_ were defined as the mean and standard deviations of the differences between offsets.

### Participants

Six adult participants (including two of the co-authors) participated in the task.

### Eye-tracking task and data recording

Following a nine-point calibration, participants viewed three images freely for 5 s each. The images depicted everyday objects (flowers, chairs) and extended approximately 7.6° horizontally and 10.3^o^ vertically, on a 23.8″ monitor. Data were recorded using a Tobii Pro Spectrum eye tracker at a sampling rate of 1200 Hz using the software Tobii Pro Lab (Tobii Inc, Danderyd, Sweden). The recorded data were of high quality, as indicated by low sample-to-sample RMSD (M = 0.12°, SD = 0.01) and little data loss (M = 1.24%, SD = 0.96%).

Data were pre-processed through linear interpolation over gaps shorter than 75 ms followed by smoothing with a moving average filter with a window size corresponding to 15 ms. Pre-processing was conducted with the kollaR function *preprocess_gaze.* The same settings were used for all event classification algorithms to facilitate comparison.

#### Comparison analysis 1: I-VT algorithm

The kollaR function algorithm_ivt was compared to fixation classification in Tobii Pro Lab (version 1.232, Tobii Inc., Danderyd, Sweden), a software for data recording and analyses sold by the eye-tracking manufacturer Tobii. The Tobii I-VT algorithm is described in Olsen (2012).

For both algorithms, we set the velocity threshold to 30°/s and the minimum duration of fixations to 60 ms and specified that subsequent fixations within a distance of 0.5° and within 75 ms should be merged. kollaR identified a total of 275 fixations, whereas Tobii Pro Lab classified 276. Fixations detected by the two algorithms had similar mean duration and standard deviations (kollaR: M = 281.09 ms, SD = 180.86; Tobii Pro Lab: M = 278.65 ms, SD = 179.19) and timing (RTO_onset_ = 0.01 ms, RTD_onset_ = 3.44 ms, RTO_offset_ = 1.35 ms, RTD_offset_ = 0.84 ms). Results from individual participants are shown in Table 2.

**Table 2:** Descriptive statistics of fixations classified by the I-VT algorithm in kollaR and Tobii Pro Lab

	kollaR (algorithm_ivt)		Tobii Pro Lab (I-VT filter)		RTO_onset_	RTD_onset_	RTO_offset_	RTD_offset_
Participant	*N*	Duration	*N*	Duration				
p01	44	305.74 (210.53)	44	304.79 (211.17)	– 0.11	1.9	0.83	0.98
p02	43	305.05 (214.01)	44	292.92 (204.85)	– 1.84	7.6	1.74	0.94
p03	42	300.81 (170.65)	42	300.02 (170.37)	0.97	3.68	1.77	0.53
p04	57	223.9 (126.19)	57	223.14 (126.92)	0.19	2.16	0.95	0.94
p05	38	343 (244.95)	38	340.46 (245.01)	– 1.16	0.98	1.38	0.71
p06	51	241.16 (83.27)	51	242.16 (83.37)	2.01	4.29	1.41	0.94
All	275	281.09 (180.86)	276	278.65 (179.19)	0.01	3.44	1.35	0.84

#### Comparison analysis 2: The adaptive velocity threshold algorithm

The kollaR function *algorithm_adaptive* was compared to fixation classification in the MATLAB implementation of the adaptive velocity algorithm presented by Niehorster et al. (2015). Both functions were run with the initial peak velocity threshold set to 200°/s, and minimum duration of fixations and saccades set to 40 ms and 12 ms, respectively. Saccades or fixations were not merged. Saccade onset and offset thresholds were defined according to the default settings described by Niehorster et al. (2015). As can be seen in Table 3, although kollaR detected a slightly higher total number of fixations (273 compared to 267), their mean durations were close to identical.

**Table 3:** Number and average duration of fixations classified by the adaptive velocity threshold algorithm in kollaR and the MATLAB implementation by Niehorster et al. (2015)

	kollaR (algorithm_adaptive)		Niehorster et al. MATLAB implementation		RTO_onset_	RTD_onset_	RTO_offset_	RTD_offset_
Participant	*N*	Duration	*N*	Duration				
p01	43	303.49 (209.94)	40	310.96 (218.84)	– 0.2	6.21	– 4.69	5.69
p02	41	269.01 (201.34)	42	270.3 (201.99)	– 1.67	7.68	– 5.54	4.58
p03	41	269.18 (166.51)	43	258.93 (171.9)	– 1.93	8.11	– 4.84	5.99
p04	60	198.33 (98.11)	54	197.31 (94.68)	– 0.18	5.87	– 3.33	5.00
p05	38	321.8 (196.26)	39	311.56 (189.56)	3.05	3.99	– 1.29	3.73
p06	50	219.13 (86.36)	49	224.93 (83.33)	4.44	4.78	3.44	1.69
All	273	257.14 (165.86)	267	257.5 (167.22)	0.59	6.11	– 2.71	4.45

### Comparison analysis 3: The I2MC algorithm

The kollaR function *algorithm_i2mc* was compared to the MATLAB implementation of the I2MC algorithm published by Hessels et al., (2017, version 2.1). In both algorithms, k-means clustering was conducted with data at the original sampling rate as well as after down-sampling with factors of 2, 5, and 10. Following the default settings in the implementation of the I2MC by Hessels et al., the threshold for k-means clustering weights was set to 2. Fixations within a distance of 0.7° and 30 ms were merged. As can be seen in Table X, kollaR detected 282 fixations with the I2MC algorithm with a mean duration of 286.83 ms (SD = 184.95). The Hessels et al. implementation of the same function detected 283 fixations (mean duration: 281.62 ms, SD = 185.78). See Table 4 for data from individual participants.

**Table 4:** Number and average duration of fixations classified by the I2MC algorithm in kollaR and the MATLAB implementation by Hessels et al., (2017)

	kollaR (algorithm_i2mc)		Hessels et al. MATLAB implementation		RTO_onset_	RTD_onset_	RTO_offset_	RTD_offset_
Participant	*N*	Duration	*N*	Duration				
p01	43	323.46 (233.18)	43	322.27 (232.88)	– 0.96	2.61	– 1.18	1.48
p02	45	298.72 (214.97)	46	286.28 (205.3)	0.9	4.1	– 2.54	3.4
p03	45	302.96 (172.18)	46	287.31 (175.03)	0.57	4.65	– 1.65	3.24
p04	58	229.55 (125.58)	57	232.13 (138.84)	– 0.46	4.27	– 1.59	2.49
p05	38	353.51 (242.72)	39	343.12 (242.69)	0.44	3.00	– 2.88	3.86
p06	53	248.17 (87.44)	52	246.99 (92.85)	– 0.7	5.38	– 2.35	2.66
All	282	286.83 (184.95)	283	281.62 (185.78)	– 0.04	4.00	– 2.03	2.86

### Conclusions

The comparison analyses demonstrate a close agreement between fixation classification algorithms in kollaR and implementations of the same algorithms in other software, including the commercially available Tobii Pro Lab. In the following sections, we demonstrate how kollaR functions can be used to compare and evaluate the performance of event classification algorithms.

## Example analysis 1: Applying and validating event classification algorithms

In this example, we illustrate how event classification algorithms can be applied using real data from individuals with neurodevelopmental conditions. For this purpose, we use data from two male participants with Fragile X syndrome, a genetic neurodevelopmental disorder characterized by mild-to-severe intellectual disability. Most males with Fragile X syndrome have an intellectual disability, as well as symptoms of inattention and hyperactivity (Hagerman et al., 2017). For these reasons, there is a significant risk of low-quality data. By visualizing and comparing the outputs of different event classification methods, we demonstrate how kollaR helps to identify potential threats to data validity.

### Participants

Two male individuals with Fragile X syndrome completed an eye-tracking task. Participant 1 was aged 34, whereas Participant 2 was 11 years old at the time of assessment. Both participants were diagnosed with intellectual disability. The study was approved by the Swedish Ethical Review Authority.

### Eye-tracking task and data recording

The data come from a task where novel stimuli appeared to the left or the right in the visual periphery while participants’ gaze was primed to the center of the screen. Participants were instructed to look freely at the images. This results in gaze data characterized by a large number of gaze shifts along the horizontal axis. Data were recorded at a sampling rate of 1200 Hz using a Tobii Spectrum eye tracker (Tobii Inc., Danderyd, Sweden). Stimuli were presented using custom scripts written in MATLAB (MathWorks, Inc.) with Psychtoolbox (Kleiner et al., 2007). Participants were seated approximately 65 cm from a 23.8-inch monitor. A five-point calibration was completed before the recording.

The proportion of valid samples for Participants 1 and 2 was 84% and 40%, respectively. This means that, whereas Participant 1 had a relatively low level of data loss, Participant 2 had a proportion of lost data that is high, but far from uncommon in research with populations with intellectual disability or other neurodevelopmental conditions. Sample-to-sample RMS was 0.20° for Participant 1 and 0.36° for Participant 2.

### Pre-processing

**Fig. 2:**
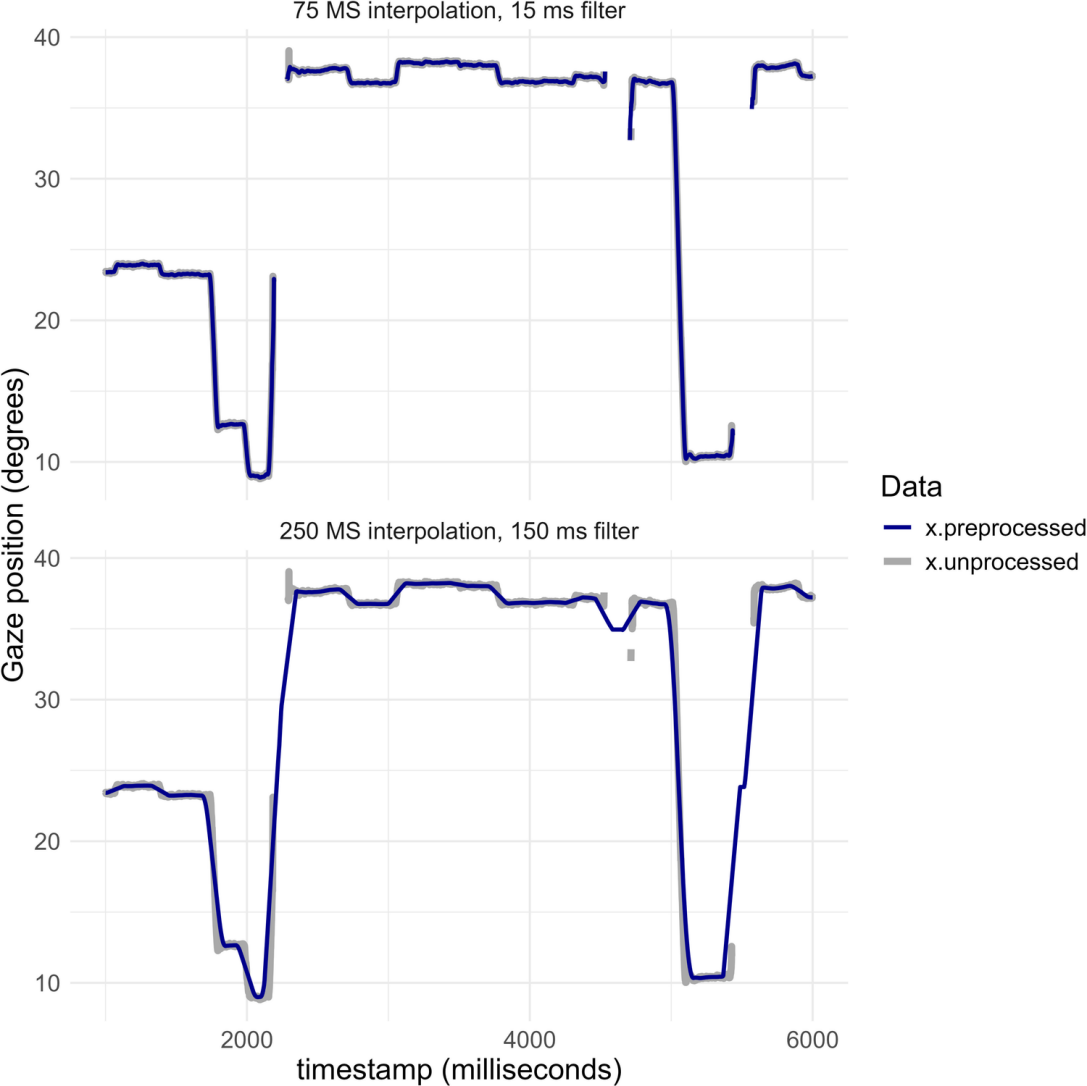
Pre-processed and unprocessed X coordinates (in degrees) during a period of approximately 5 s. *Top* A relatively conservative pre-processing with interpolation of gaps in the data shorter than 75 ms followed by moving average smoothing with a window of 15 ms. *Bottom* A liberal pre-processing with an interpolation window increased to 250 ms and moving average window increased to 150 ms. Pre-processing was performed with the kollaR function preprocess_gaze. The default settings were used in the upper panel. The figure was created with the function fixation_plot_ts

The first step in the analysis is pre-processing, which includes blink removal and smoothing. The function *preprocess_gaze* in kollaR achieves this through linear interpolation over gaps in the data and subsequent smoothing of the *X* and *Y* coordinates with a moving median filter. Interpolation is based on a segment of data before and after the gap to avoid short bursts of noise close to the gap. Default settings are described in (Olsen, 2012). The choice of pre-processing settings can have a significant impact on the data, as can be seen in Fig. 2, which shows unprocessed and processed *X* coordinates from a period including several gaps following two different pre-processing procedures. A relatively conservative pre-processing (Fig. 2, upper panel) interpolation of gaps in the data with a maximum length of 75 ms followed by moving average smoothing with a window corresponding to 15 ms) produces a variable that follows the original data closely. A more liberal pre-processing (Fig. 2, lower panel, interpolation of gaps with a maximum length of 250 ms followed by moving average filtering with a window corresponding to 150 ms) recovers more data but increases the risk of artifacts. Specifically, long interpolated gaps may create artificial patterns, such as brief fixations, that were not present in the original recording. This can mislead event classification algorithms into detecting false fixations or saccades. Secondly, the filter with the longer time window makes rapid shifts in gaze position to appear less abrupt and induces an apparent small shift in their timing. Visualizations like these can help researchers compare the effects of different pre-processing procedures on their data. Figure 2 was produced with the kollaR function *fixation_plot_ts.*

### Comparing fixation classification algorithm output and raw data

**Fig. 3:**
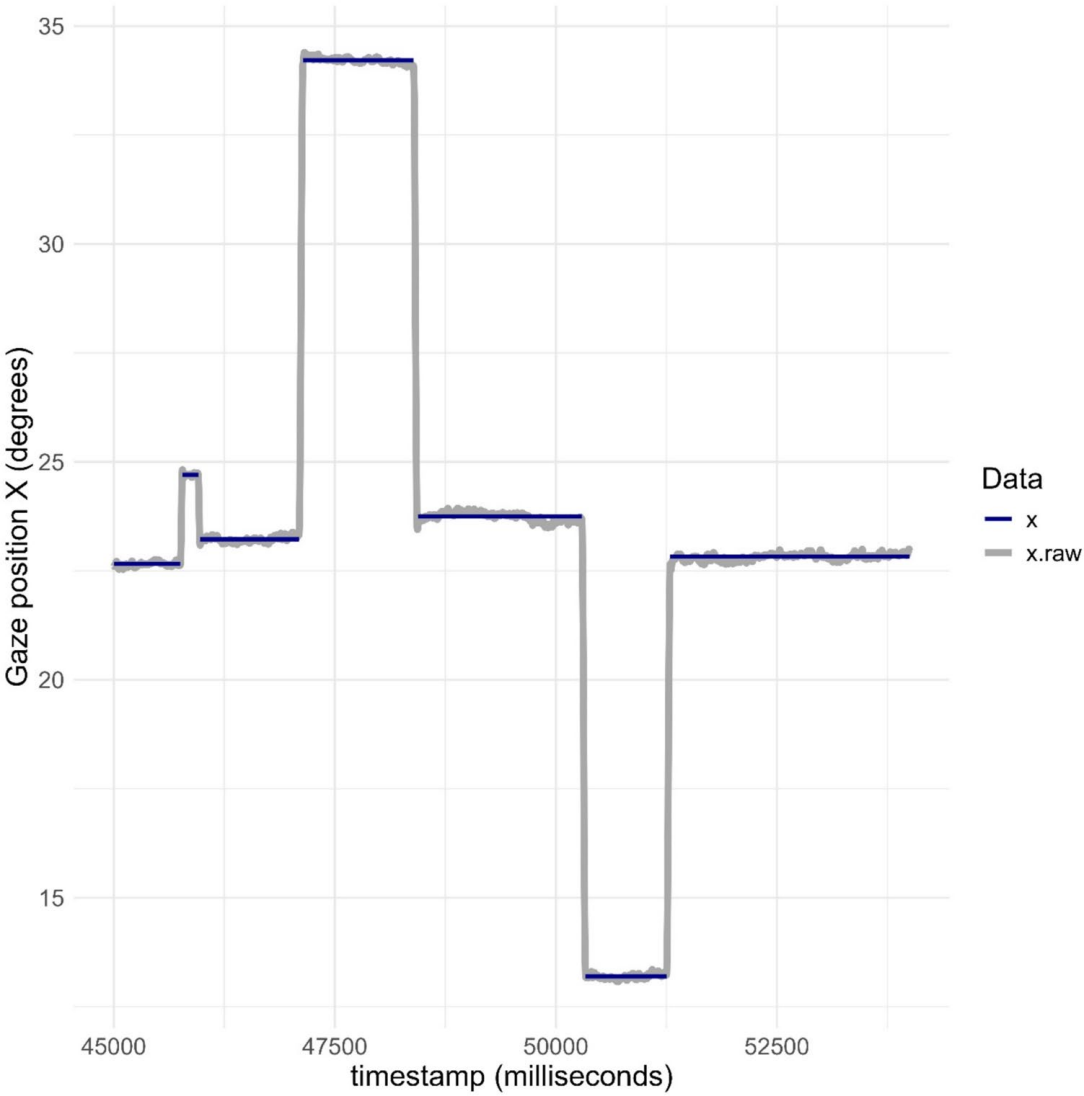
Raw (“x.raw”) and fixation classified (“x”) during a time segment of approximately 6.25 s. The plot was generated with the function fixation_plot_ts

Visualizations of single sample and fixation coordinates in the same plot can be useful for evaluating the output of classification algorithms. kollaR includes several functions to facilitate this process. Figure 3 shows raw and fixation *X* coordinates from participant 1 during a time segment of approximately 6.25 s. Fixations were classified with the function *algorithm_ivt* with the velocity threshold set to 30°/s. The plot was generated using the function *fixation_plot_temporal.* The *X* coordinate vector (labeled “gaze position”) includes periods of relatively stable gaze positions interleaved with quick displacements representing fixations and saccades, respectively. The fixation classification algorithm seems to function adequately during this time segment.

**Fig. 4:**
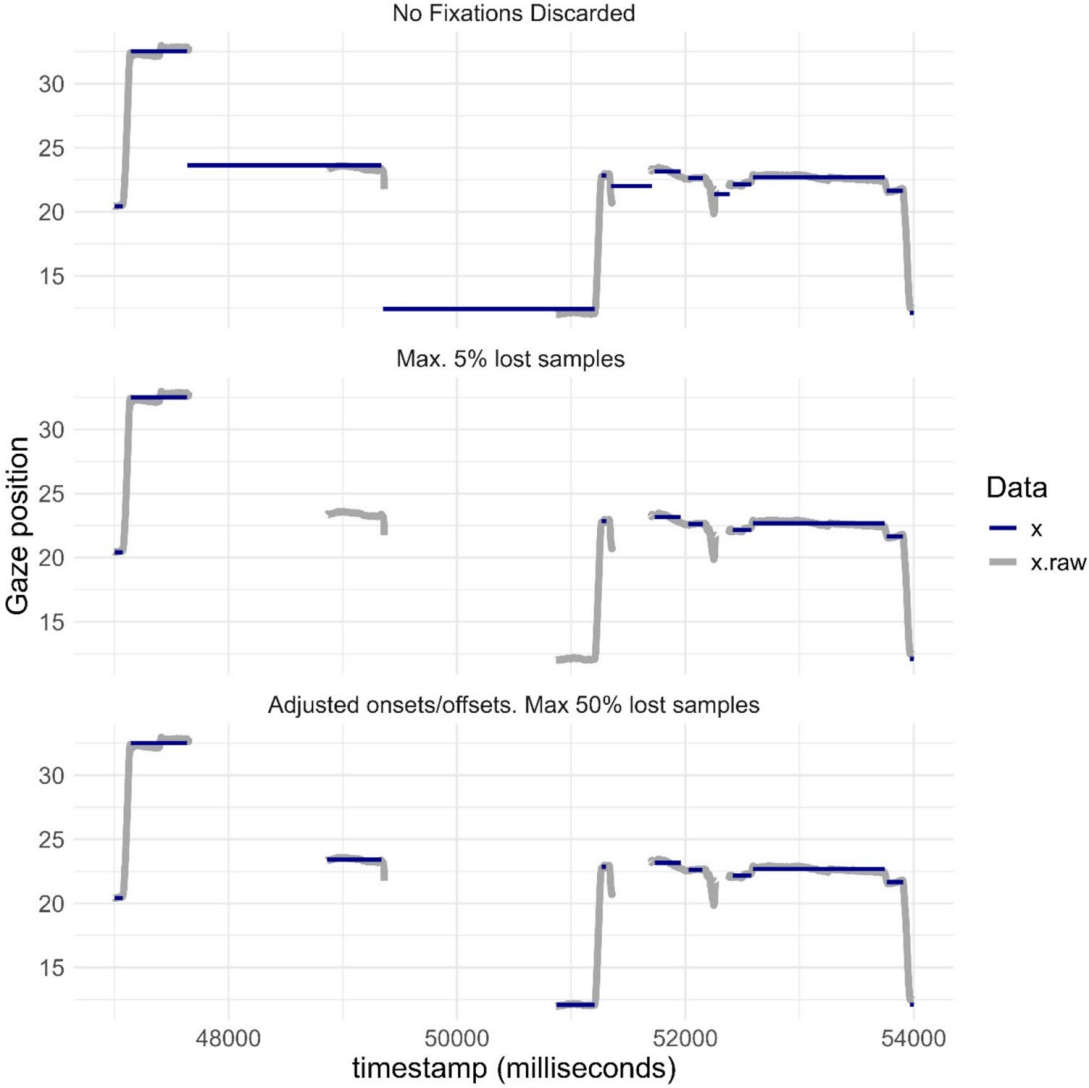
Fixation classified and raw *X*-coordinates (in degrees) from participant 2. Data were sampled at 1200 Hz. Fixations were identified using the I-VT algorithm with a velocity threshold set to 30°/s. Timestamps are shown in milliseconds. Event classification was conducted using the kollaR function *algorithm_ivt*. The plot was generated with the function *fixation_plot_ts*

Joint visualizations of raw and event-classified data can also identify problems with the analysis pipeline. By default, the I-VT algorithm classifies periods between saccades as belonging to fixations. If the algorithm is not completed with a rule that discards periods with very little data, there is a risk of incorrect fixation classification. The kollaR event classification algorithms exclude fixations with a high proportion of lost gaze by default. Figure 4 shows a time segment from Participant 2 during approximately 2 s with this option turned off. Event detection was conducted with an I-VT algorithm with velocity set to 30°/s to the data. This figure indicates a problem with the fixation classification algorithm. Several fixations are “detected” during periods with little or no actual data. Here, the rapid shifts in gaze position right before the gaps have been treated as saccades. In our experience, artefacts like these are not uncommon in low-quality data from individuals with neurodevelopmental conditions, and researchers should take care to avoid them influencing their results. kollaR includes several potential solutions to this problem. These include removal of fixations with a high number of invalid samples (this can be done by changing the parameter *missing.samples.threshold* in the function *algorithm_ivt*) and adapting the velocity threshold parameter.

### Examining the impact of parameter settings on fixation classification

**Fig. 5:**
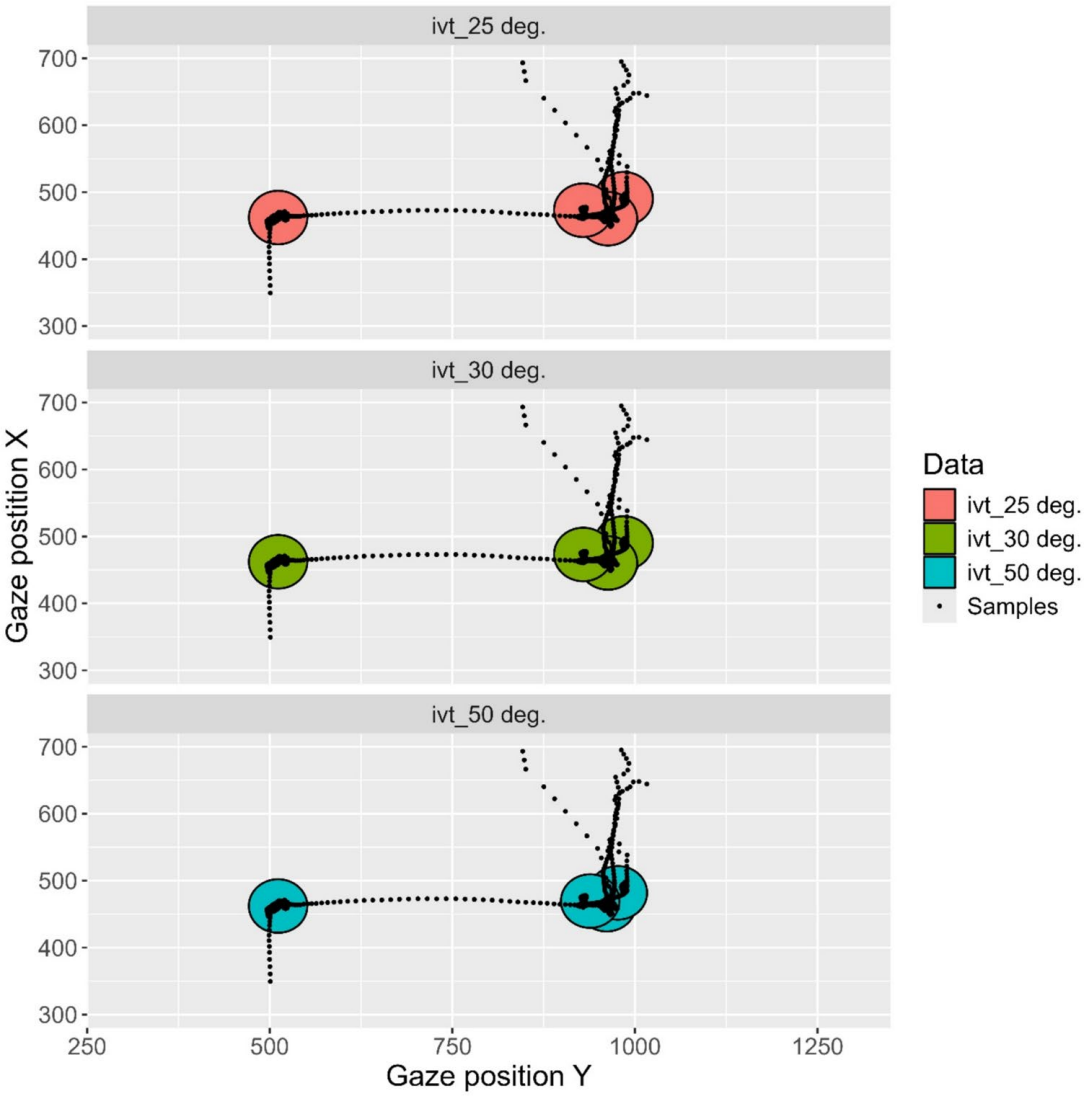
Fixations of classified by the I-VT algorithm at different velocity threshold values. Data from Participant 1. Gaze position is shown in pixels

Visualizations are helpful for selecting event classification algorithms. Figure 5 shows the fixations identified by the I-VT algorithm using three different threshold parameter values in a segment of data from Participant 1. Data are plotted in a 2D space. The plot was generated by the kollar function *filt_plot_2d*. Reassuringly, the three threshold values lead to consistent results in this data segment. If results look similar for a range of different sub-sections of the data set, it suggests that the choice of algorithm is not affecting the results much.

**Fig. 6:**
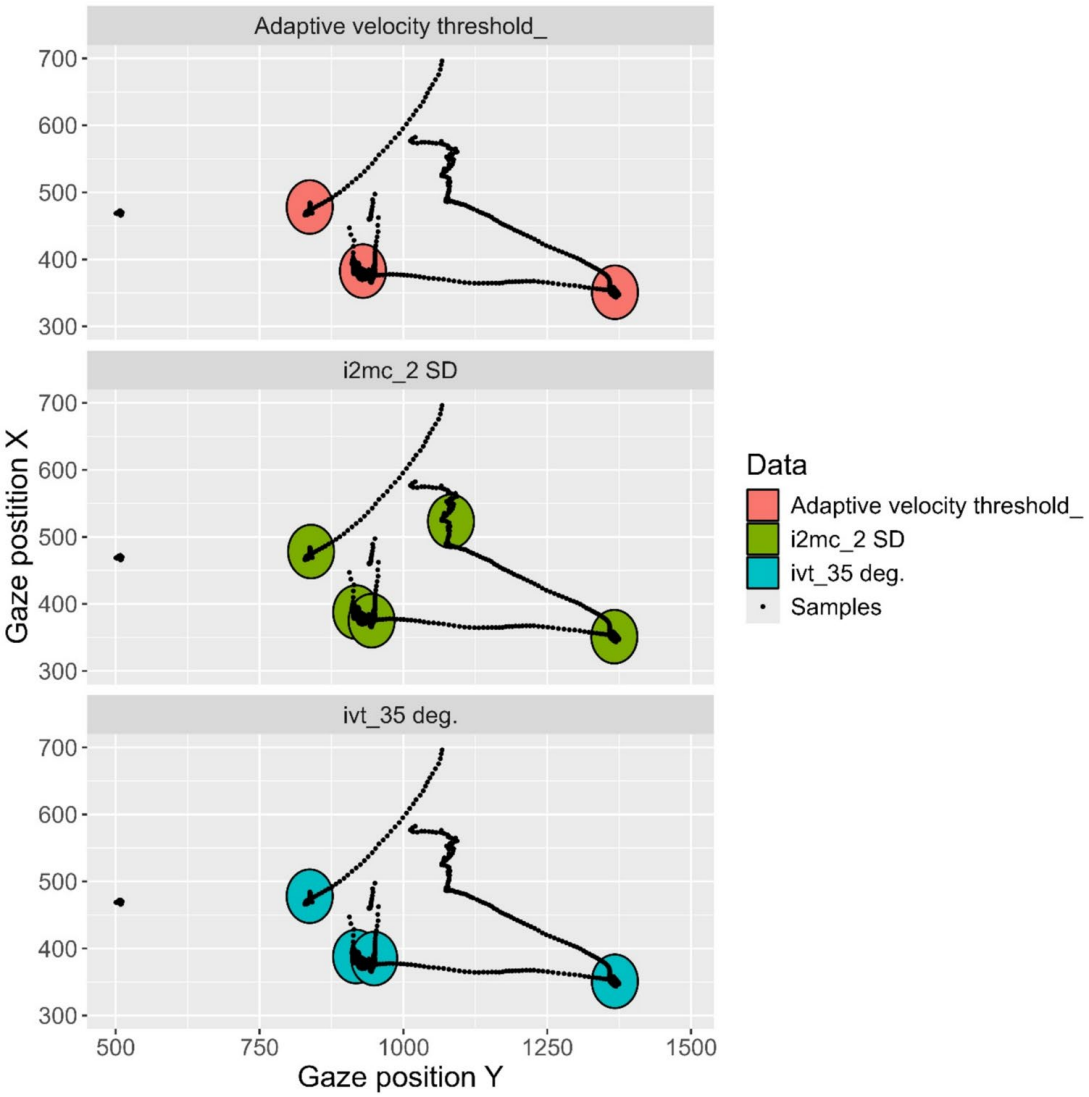
Fixations identified by the I2MC and I-VT algorithms in a time segment from Participant 2. Gaze position is shown in pixels

Figure 6 shows the fixations detected by two different algorithms during a period of data from Participant 2. Although results are similar, the I2MC algorithm reports a fixation in the upper part of the plot which is not reported by the I-VT algorithm. Visualizations like these over a range of data segments can indicate systematic differences between algorithms. This would merit further examination before selecting an algorithm. It would be important to examine whether the discrepancies between algorithms are more likely to be found in time segments characterized by noisy data. Researchers examining group differences (for example, in fixation time between autistic and non-autistic children) would be interested in whether data from both groups are equally sensitive to differences between algorithms. It would also be of interest to know which outcome measures these discrepancies could affect. For example, do the algorithms disagree on the number of fixations, but not on their total duration or location? This could indicate a problem for a study that examines the number of fixations, but not necessarily pose a threat to an analysis of total fixation duration.

### Comparing fixation metrics between candidate algorithms

**Fig. 7:**
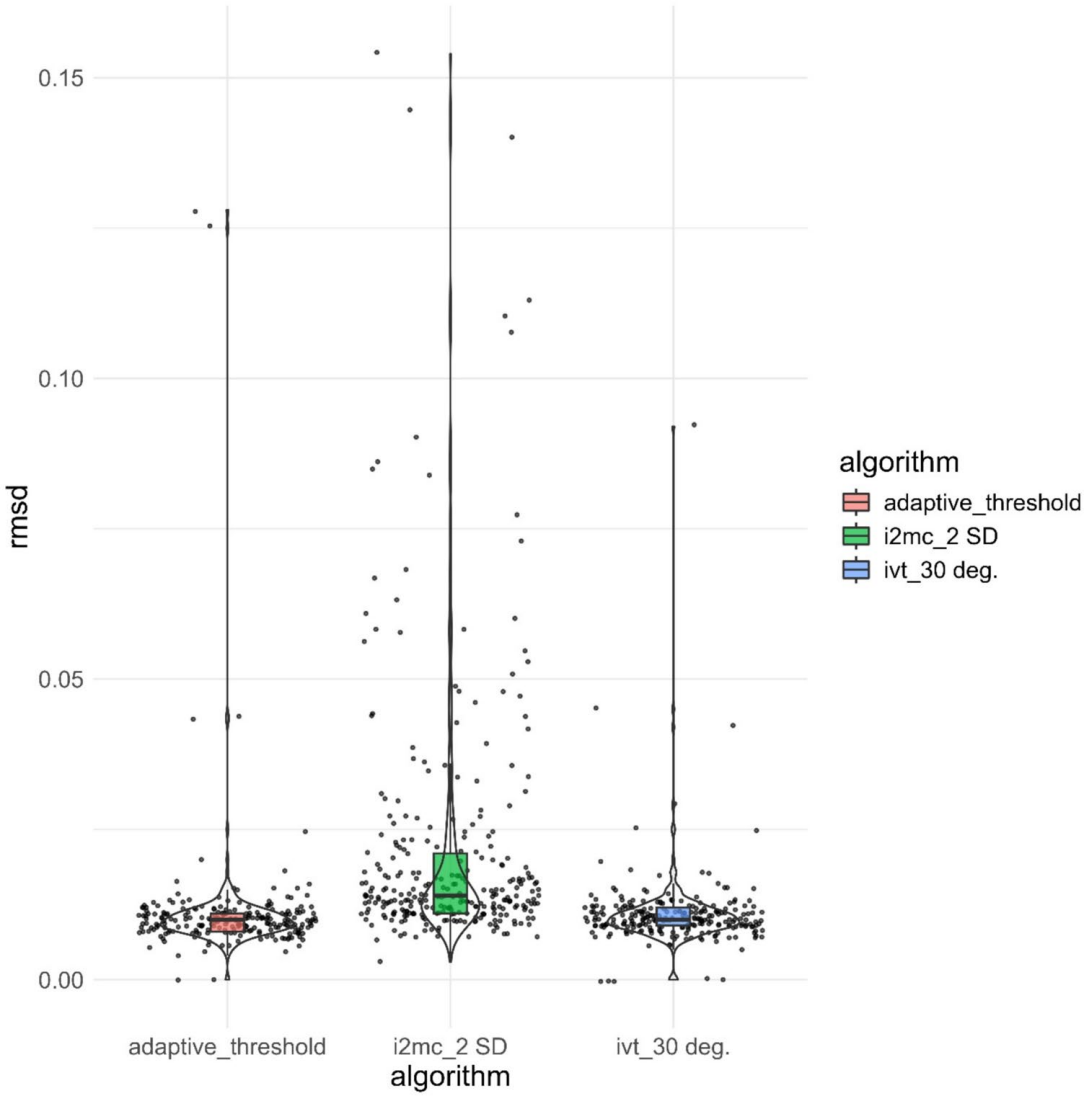
RMSD values of fixations detected by the I-DT (dispersion), I2MC, and I-VT algorithms. Data from Participant 1

So far, we have shown how plots comparing raw and filtered data over selected time intervals can be used to examine the outcomes of event classification algorithms. It is also useful to compare aggregated measures of fixations. The function *plot_algorithm_results* visualizes quality metrics from one or several event classification algorithms, including the proportion of data loss and the sample-to-sample root mean squared deviations (RMSD, e.g., *precision*) of all included raw samples. High RMSD values or proportion of missing samples indicate low data quality (see Duchowski, 2017 for a discussion). Figure 7 shows the RMSD of three candidate fixation algorithms for Participant 1. As can be seen, the I-VT and adaptive velocity algorithms both identify fixations with lower RMSD values than the I2MC algorithm. This indicates that the former algorithms classified fixations in data with higher data quality. If the data is generally of high quality, it could be argued that the results shown in Fig. 7 support the use of the I-VT or adaptive velocity threshold algorithms over the I2MC. However, studies of young children or participants with neurodevelopmental conditions or infants will often result in low-quality data. In this case, it may be preferable to use a classification algorithm such as the I2MC rather than discarding large parts of the data (for a discussion, see Hessels et al., 2017).

### Merging short fixations?

**Fig. 8:**
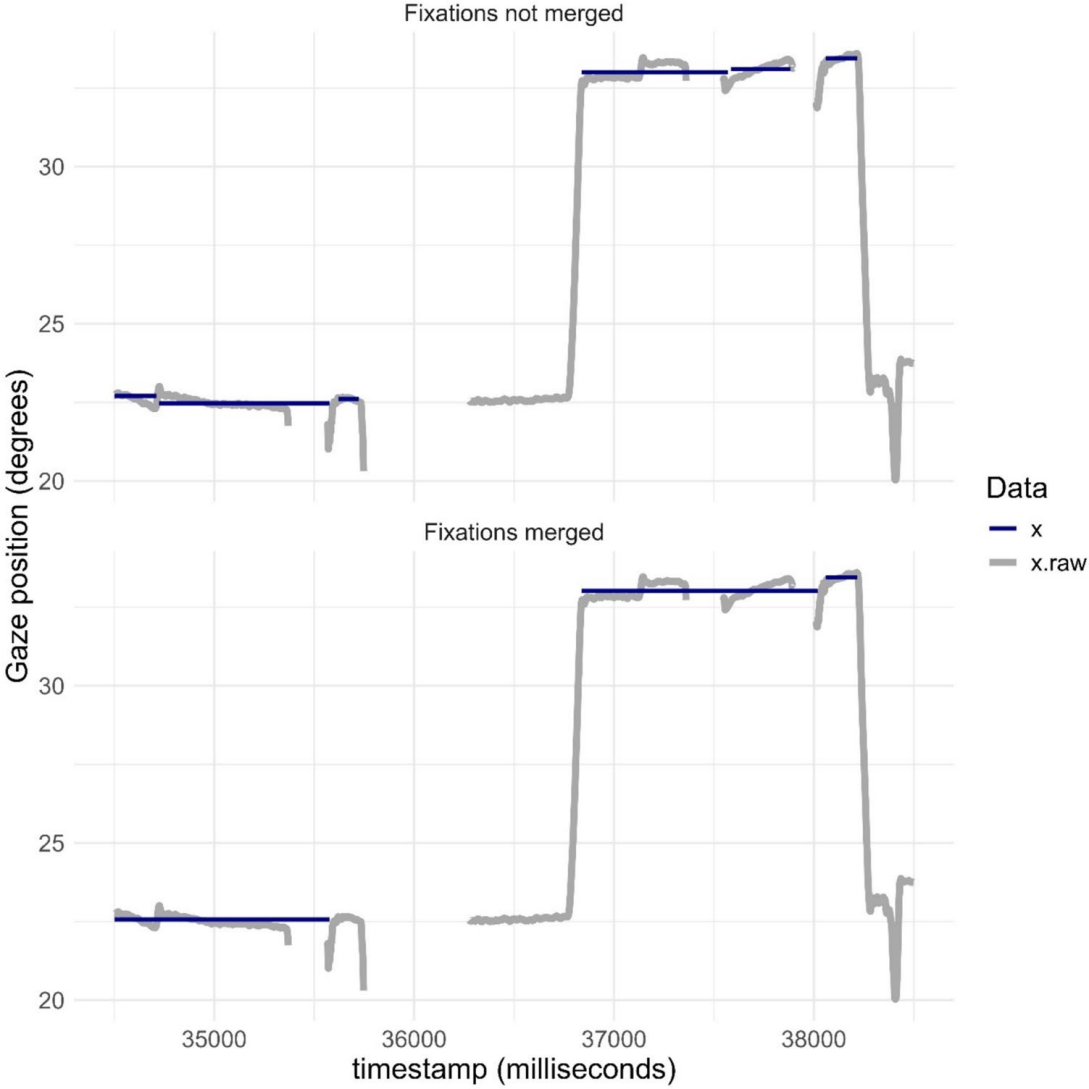
Raw and fixation classified *X* coordinates (“gaze position”) without (**A**) and with (**B**) merging of adjacent fixations. Data from Participant 2. Timestamps in milliseconds

Short periods of lost samples or low precision may cause fixation classification algorithms to split longer fixations into several shorter ones. It is common to compensate for this by merging fixations that are very close in space and time (e.g., Olsen, 2012). In a hypothetical case, a researcher finds that autistic children make more fixations when looking at faces than non-autistic children, despite similar overall fixation time. At the same time, the proportion of lost data is higher in the autistic group. Are these results evidence for differences in cognitive processing? Or are they merely an artefact caused by noise in the data? It can be difficult to answer this question without a detailed description of how the data were processed, including the event classification algorithm and the criteria (if any) for merging fixations. kollaR makes it possible to use event classification algorithms both with and without merging of fixations and to compare results. Figure 8A shows a data segment from Participant 2 without fixation merging. As can be seen, three short fixations at close distance from each other on the *X*-axis are detected before a saccade (indicated by a rapid change in gaze position), followed by three additional short fixations. The same data are shown in Fig. 8B, this time merging subsequent fixations occurring closer than 1.5° and within 200 ms of each other. In this case, only three fixations remain in the entire time segment, although the total fixation time is identical. If the number of fixations is important in the analysis, the decision to merge adjacent fixations or not (and if, at what distance) could have a dramatic effect on the data. This decision will ultimately depend on the study design and research question as well as on operationalizations of what a fixation is (Hooge et al., 2022).

### What is an adequate velocity threshold for the velocity threshold algorithm?

Selecting the velocity threshold for saccade classification can be challenging. If the threshold is set too low, there is a risk that longer fixations are split into several short ones (Olsen & Matos, 2012). On the other hand, too high a threshold may cause small saccades to be missed, leading to overestimation of fixation length. Importantly, there is no optimal threshold value for all potential data sets, but instead, the threshold should be evaluated in the light of factors such as data quality and research questions (Hooge et al., 2022; Salvucci & Goldberg, 2000). The function *plot_sample_velocity* plots the sample-to-sample velocity of a data segment and a proposed threshold for saccade identification (an example is shown in Fig. 1). This can facilitate an analysis of the effects of potential thresholds on the output. As noted above, the adaptive velocity threshold algorithm, which determines a threshold based on data properties, is an alternative to manual threshold selection.

## Example analysis 2: Analyzing data and creating plots for publications and presentations

### Analyzing the results and creating plots for publications and presentations

Once the fixation classification algorithm has been selected, validated, and implemented, the next step is to analyze the data. kollaR can be used to conduct area of interest (AOI) based analyses, such as examining fixation time, the number of fixations, and latency to first fixation or saccade entering and/or leaving an AOI during the whole recording or segments of the data (i.e., trials during an experiment). These analysis methods are sufficient for the most common research designs in developmental science in areas such as autism, anxiety disorders, and infant development (e.g., Falck-Ytter et al., 2023; Günther et al., 2021; Lisk et al., 2019; Setien-Ramos et al., 2023). In the following example, we demonstrate how kollaR can be used to visualize individual and group-level data.

#### Participants and eye tracking task

Seven student volunteers viewed images of the paintings *Angelus Novus* and *Senecio* by Paul Klee presented side by side for 6 s. Participants were asked to view the stimuli freely. Stimuli were presented on a 23.4-inch monitor, and data were recorded at 1200 Hz using a Tobii Pro Spectrum following a nine-point calibration. The data are here arbitrarily split into two groups.

#### Visualizing the results and analyzing fixation and saccade metrics

kollaR includes functions for drawing and saving AOIs on stimulus images, and summarizing saccade and fixation metrics during selected time segments. These include total fixation duration, the number of fixations or saccades, and the latency to a first gaze event inside or outside an AOI.

**Fig. 9:**
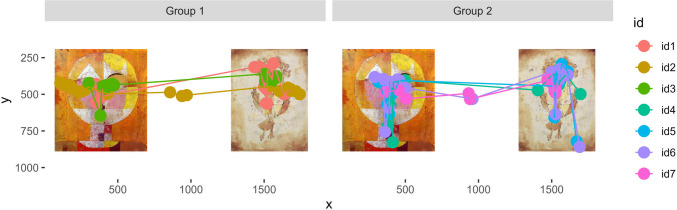
Example of a scanpath plot. The figure shows the scanpaths of seven participants during 6 s. Participants were arbitrarily split into two groups

It is often useful to visualize the data in publications and presentations. The kollaR function *static_plot* visualizes fixations during a selected time period for individual participants or groups. The function *animated_fixation_plot* takes the same arguments as *static_plot* and produces a dynamic.gif video which shows gaze behavior in real time. The stimulus images shown during the task can be added as a background. Figure 9 shows an example of a static plot.

## Conclusion

Classification of eye-tracking data as fixations and saccades is an important part of many eye-tracking analyses. The way these are conducted can have a huge impact on the results, yet is often insufficiently described in published papers. kollaR is an open-source R package for the analysis and visualization of eye-tracking data. We have demonstrated how kollaR can be used to perform eye-tracking analyses, from pre-processing and fixation classification to summarizing results and visualization. Specifically, kollaR can be used to visualize how methodological choices affect each step of the analysis. This can, in turn, help researchers select appropriate fixation classification algorithms. It is possible to use kollaR in all or some of the analytic steps. For example, kollaR can be used to classify fixations in raw data or to evaluate fixation classification implemented in software provided by eye-tracker manufacturers, as long as the data can be read into R. Researchers analyzing gaze data without explicitly considering fixations or saccades can use kollaR to visualize their data and identify potential artifacts in their analysis pipeline. Finally, kollaR can also be used to create figures for publication after pre-processing and event classification in other software.

In its current version, kollaR includes a number of different algorithms for event classification. In a validation analysis, we compared fixation classification in kollaR to previously published software implementations of three of these algorithms (I-VT, I2MC, NH2010). This analysis yielded highly consistent results, supporting the validity of kollaR for fixation classification.

Quite often, data from participants with neurodevelopmental conditions have elevated levels of spatial noise and gaps in the data time-series; hence, these data are particularly sensitive to artifacts produced by incorrect data processing. Using data from typically developed individuals as well as two individuals with neurodevelopmental conditions (Fragile X syndrome and intellectual disability), we have demonstrated how kollaR can be used to identify and address some of these problems.

In its current version, kollaR can be used to conduct many common types of eye tracking analyses. However, it does not include functions for analyzing microsaccades, smooth pursuit of moving stimuli, or fixation classification of data from eye-tracking glasses that record gaze in 3D space rather than on a screen. To sum up, kollaR can facilitate eye tracking analyses by providing researchers with flexible tools for each step of the analytic pipeline from raw data to publication-ready figures.

## Data Availability

The data used in example analysis two are included in the kollaR package, which can be accessed through the CRAN repository (see https://CRAN.R-project.org/package=kollaR). The data used in example analysis 1 is not made public due to data protection regulations.

## Footnotes

1 *Kolla* is a Swedish word meaning “look”.

## Code availability

The kollaR code is open source and available from the CRAN repository (see https://CRAN.R-project.org/package=kollaR).

## Declarations

### Open Practices Statement

No analyses were pre-registered.
